# Id4 dependent acetylation restores mutant-p53 transcriptional activity

**DOI:** 10.1186/1476-4598-12-161

**Published:** 2013-12-13

**Authors:** Ashley E Knowell, Divya Patel, Derrick J Morton, Pankaj Sharma, Shanora Glymph, Jaideep Chaudhary

**Affiliations:** 1 Center for Cancer Research and Therapeutics Development, Clark Atlanta University, 223 James P. Brawley Dr. SW, Atlanta, GA 30314, USA

**Keywords:** Id4, p53, Acetylation, CBP/p300, Prostate, DU145

## Abstract

**Background:**

The mechanisms that can restore biological activity of mutant p53 are an area of high interest given that mutant p53 expression is observed in one third of prostate cancer. Here we demonstrate that Id4, an HLH transcriptional regulator and a tumor suppressor, can restore the mutant p53 transcriptional activity in prostate cancer cells.

**Methods:**

Id4 was over-expressed in prostate cancer cell line DU145 harboring mutant p53 (P223L and V274F) and silenced in LNCaP cells with wild type p53. The cells were used to quantitate apoptosis, p53 localization, p53 DNA binding and transcriptional activity. Immuno-precipitation/-blot studies were performed to demonstrate interactions between Id4, p53 and CBP/p300 and acetylation of specific lysine residues within p53.

**Results:**

Ectopic expression of Id4 in DU145 cells resulted in increased apoptosis and expression of BAX, PUMA and p21, the transcriptional targets of p53. Mutant p53 gained DNA binding and transcriptional activity in the presence of Id4 in DU145 cells. Conversely, loss of Id4 in LNCaP cells abrogated wild type p53 DNA binding and transactivation potential. Gain of Id4 resulted in increased acetylation of mutant p53 whereas loss of Id4 lead to decreased acetylation in DU145 and LNCaP cells respectively. Id4 dependent acetylation of p53 was in part due to a physical interaction between Id4, p53 and acetyl-transferase CBP/p300.

**Conclusions:**

Taken together, our results suggest that Id4 regulates the activity of wild type and mutant p53. Id4 promoted the assembly of a macromolecular complex involving CBP/P300 that resulted in acetylation of p53 at K373, a critical post-translational modification required for its biological activity.

## Introduction

Id (Inhibitor of differentiation) proteins (Id1, Id2, Id3 and Id4) are dominant negative regulators of basic helix loop helix transcription factors such as TCF3 [1,2]. Apart from blocking the general bHLH-DNA (E-box response element) interactions, the Id1, 2 and 3 proteins also interact with several non-bHLH proteins such as CASK, ELK1, 3 and 4, GATA4, caveolin, CDK2, PAX2, 5 and 8, Rb and related pocket proteins and ADD1 ( [1,2] and public databases). Currently, the non-bHLH interaction partners for Id4 are not known. Id proteins can thus control many cellular processes such as cell growth, differentiation, and apoptosis [3], through specific bHLH and non-bHLH interactions.

Id proteins in general, promote proliferation and inhibit differentiation with few exceptions such as Id2 and Id4 that can also promote differentiation in some organ systems. Id4 promotes differentiation of osteoblasts [4], adipocytes [5], neurons [6], but inhibits oligodendroglial differentiation [7] by blocking the transcriptional activity of bHLH protein Olig1/2.

Majority of studies have demonstrated tumor suppressor activity of Id4 which is largely based on the evidence that it is epigenetically silencing in cancers such as leukemia [8], breast [9,10], colorectal [11] mouse and human CLL (chronic lymphocytic leukemia [12]) and gastric cancer [13]. High Id4 expression is observed in bladder [14] and rat mammary gland carcinomas, [15], whereas chromosomal translocation of Id4 (t(6;14)(p22;q32)) was found in B-cell acute lymphoblastic leukemia [16] and B-cell precursor acute lymphoblastic leukemia (BCP-ALL) [17], suggesting that it may also have tumor promoter activity.

Decreased Id4 expression with increasing grade of prostate cancer is also associated with Id4 promoter hyper-methylation [18,19]. The prostate cancer cell line DU145 also lacks Id4 expression due to promoter hypermethylation whereas LNCaP cells express Id4 [20]. Interestingly, DU145 cells also harbor mutant p53 with extended half-life, a property associated with mutated forms of p53 [21]. The p53 mutants (P223L and V274F) in DU145 cells are rare but located within the DNA binding domain (DBD 94-292) known to abrogate p53 activity [22,23]. The V274F mutation in DU145 cells is next to R273H/C/L/P, a DNA contact and one of the most highly mutated amino acid in p53 [23]. Both these amino acids (274°F and 273H) are within the conserved region of p53 beta strand S10 whereas 223 L lies in the outer loop [24]. Studies have shown that some but not all p53 mutations maintain transactivation potential for some promoters (e.g. CDKN1a) but not others (e.g. BAX, PUMA and Pig3) [25]. Likewise, the mutant p53 in DU145 also lacks the ability to trans-activate CDKN1A [21]. We have shown that ectopic expression of Id4 in DU145 cells triggers apoptosis and CDKN1A dependent cell cycle arrest [20]. CDKN1A being a prototype p53 transcriptional target prompted us to investigate whether Id4 promoted mutant p53 transcriptional activity in DU145 cells. The results presented in this study demonstrate that Id4 can promote the binding of mutant p53 to its response element on the p21 promoter and other p53 responsive apoptotic target genes such as BAX and PUMA. At the mechanistic level we demonstrate that Id4 recruits acetyl transferase CBP/p300 to promote acetylation of p53. Thus, mutant p53 in DU145 may retain conformational flexibility which upon post-translational modification could achieve wild type activity. Studies reported earlier have indeed shown that PCAF dependent acetylation can restore wild type activity of certain p53 mutants (G245A and R175H) [26]. Since more than one third of prostate cancers harbor mutant p53 [27,28] and majority of prostate cancers also lack Id4 [18,19]; hence physiological mechanisms involved in the transition of mutant p53 to wild type activity are of clinical relevance.

## Materials and methods

### Id4 over-expression and silencing in prostate cancer cell lines

LNCaP, DU145 and PC3 prostate cancer cell lines were purchased from ATCC and cultured as per ATCC recommendations. Human Id4 was over-expressed in DU145 cells as previously described [20]. Id4 was stably silenced in LNCaP cells using gene specific shRNA retroviral vectors (Open Biosystems #RHS1764-97196818,-97186620 and 9193923 in pSM2c, termed as Id4shRNA A, B and C respectively). The cells transfected with non-silencing shRNA (RHS1707) was used as control. Transfections and selection of transfectants (puromycin) were performed as suggested by the supplier. Successful Id4 gene silencing was confirmed by qRT-PCR and Western blot analysis.

### Western blot analysis and Co-immunoprecipitation

30 μg of total protein, extracted from cultured prostate cancer cell lines using M-PER (Thermo Scientific) was size fractionated on 4-20% SDS-polyacrylamide gel (5% for CBP/p300 western blotting). The SDS-gel was subsequently blotted onto a nitrocellulose membrane (Whatman) and subjected to western blot analysis using respective protein specific antibodies (Additional file 1: Table A1). After washing with 1× PBS, 0.5% Tween 20, the membranes were incubated with horseradish peroxidase *(*HRP*)* coupled secondary antibody against rabbit IgG and visualized using the Super Signal West Dura Extended Duration Substrate (Thermo Scientific) on Fuji Film LAS-3000 Imager.

To detect the protein-protein interactions, co-immunoprecipitation was performed using protein A coupled to magnetic beads (Protein A Mag beads, GenScript) as per manufacturer’s instructions. Briefly, protein specific IgG (anti-p53 or-Id4, Additional file 1: Table A1) was first immobilized to Protein A Mag Beads by incubating overnight at 4°C. To minimize the co-elution of IgG following immuno-precipitation, the immobilized IgG on protein A mag beads was cross-linked in the presence of 20 mM dimethyl pimelimidate dihydrochloride (DMP) in 0.2 M triethanolamine, pH8.2, washed twice in Tris (50 mM Tris pH7.5) and PBS followed by final re-suspension and storage in PBS. The cross-linked protein specific IgG-protein A-Mag beads were incubated overnight (4C) with freshly extracted total cellular proteins (500 μg/ml). The complex was then eluted with 0.1 M Glycine (pH 2-3) after appropriate washing with PBS and neutralized by adding neutralization buffer (1 M Tris, pH 8.5) per 100 μl of elution buffer.

### Chromatin immuno-precipitation (ChIP) assay

Chromatin immuno-precipitation was performed using the ChIP assay kit (Millipore, Billerica, MD) as per manufacturer’s instructions. The chromatin (total DNA) extracted from cells was sheared (Covaris S220), subjected to immuno-precipitation with p53, normal IgG or RNA pol II antibodies (Additional file 1: Table A1), reverse cross linked and subjected to qRT-PCR in Bio-Rad CFX. The previously published CHiP primer sets spanning the consensus p53 response element sites in the promoters of BAX [29], p21 [29], PUMA [30] and MDM2 [29] were used (Additional file 2: Table A2). The first intron of TCF3 (E2A) was used a negative control for p53 ChIP assays (Additional file 2: Table A2). The lack of consensus p53 response element was confirmed by subjecting the TCF3 intron 1 sequence to TRANSFAC database search [31].

### Quantitative real time PCR (qRT-PCR)

qRT-PCR was performed as described previously using gene specific primers (Additional file 2: Table A2) on RNA purified from cell lines [32].

### Electrophoretic mobility shift assay *(*EMSA*)*

The nuclear proteins from respective cell lines were prepared using the nuclear extraction kit from Affymetrix (AY2002) as per manufacturer’s instructions. 1 μg of nuclear proteins were used in an EMSA reaction using Biotin end labeled p53 double stranded oligonucleotide (Affymerix, AY1032, p53(1) EMSA kit containing the p53 response element [29]: 5′-TAC AGA ACA TGT CTA AGC ATG CTG GGG ACT. The biotin end labeled mutated p53 response element (5′-TAC AGA ATC GCT CTA AGC ATG CTG GGG ACT) was used as a negative control. The nuclear proteins and labeled oligonucleotide or excess unlabeled oligonucleotide were incubated for 20mins at room temperature, separated on 5% non-denaturing polyacrylamide gel and transferred onto nitrocellulose membrane and detected following manufacturer’s instructions. The EMSA using LNCaP cells with wild type p53 and p53 null PC3 was used as positive and negative controls respectively.

### P53 activity assay

p53 DNA binding activity and quantitation on nuclear extracts was performed by capturing p53 with double stranded oligonucleotides containing a p53 consensus binding site immobilized in a 96 well format (TF-Detect p53 Assay, Genecopoeia) followed by detection with p53 specific antibody in a sandwich ELISA based format as per manufacturer’s instructions (essentially a quantitative super-shift assay).

### Transient transfections and reporter gene assay

Cells were cultured in 96-well plates to 70-80% confluency and transiently transfected by mixing either PG13-luc (containing 13 copies wt p53 binding sites [33], Addgene) or MG15-luc (containing 15 mutant p53 binding sites [33], Addgene) with pGL4.74 plasmid (*hRluc/TK*: Renilla luciferase, Promega) DNA in a 10:1 ratio with FuGENE HD transfection reagent (Promega) in a final volume of 100 ul of Opti-MEM and incubated for 15 min at room temperature. The transfection mix was then added to the cells. After 24 h, the cells were assayed for firefly and Renilla luciferase activities using the Dual-Glo Luciferase reporter assay system (Promega) in LUMIstar OPTIMA (MHG Labtech). The results were normalized for the internal Renilla luciferase control.

### Immuno-cytochemistry

Cells were grown on glass chamber slides up to 75% confluency. The slides were then washed with PBS (3x) and fixed in ice cold methanol for 10 min at room temperature and stored at-20°C until further use. Before use, the slides were equilibrated at room temperature, washed with PBS (5 min ×3), blocked with 1%BSA in PBST for 30 min at room temp and Incubated overnight (4C) with primary antibody (1% BSA in PBST, Additional file 1: Table A1). The slides were then washed in PBS and incubated with secondary antibody with fluorochrome conjugated to DyLight (Additional file 1: Table A1) in 1% BSA for 1 hr at room temp in dark. The slides were subsequently washed again and stained in DAPI (1 μg/ml) for 1 min and mounted with glycerol. Images were acquired by Zeiss fluorescence microscope through Axiovision software.

### Apoptosis assay and mitochondrial membrane potential (MMP)

Apoptosis and MMP was quantitated using Propidium Iodide, Alexa Fluor 488 conjugated Annexin V (Molecular Probes) and dual-sensor MitoCasp (Cell Technology) respectively, as described previously [34].

### Statistical analysis

Quantitative real time data was analyzed using the ΔΔCt method. The CHiP data was analyzed using % chromatin (1%) as input (Life Technologies). Within group Student’s t-test was used for evaluating the statistical differences between groups.

## Results

### Generation of Id4 expressing and non-expressing prostate cancer cell lines

**Figure 1:**
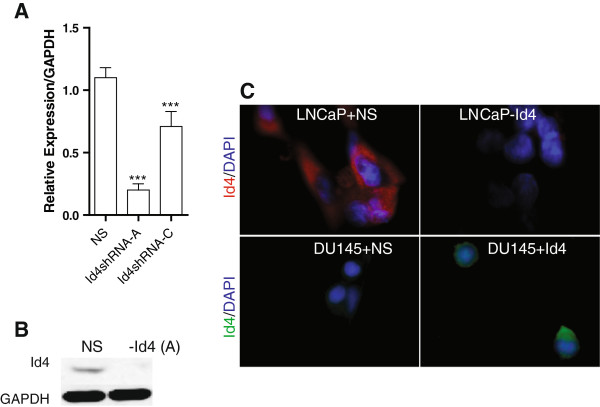
**Stable knockdown of Id4 by retroviral shRNA in LNCaP cells (retroviral vectors A and C) and stable over-expression of hId4 in Du145 cells. A**. Real time quantitative polymerase chain reaction for Id4 expression in LNCaP (NS, non-specific) following transfection with Id4 shRNA vectors A and C and non-silencing shRNA (NS) (***: P < 0.001). **B**. Western blot analysis of Id4 expression in LNCaP cells with non-specific shRNA (NS) and Id4 specific shRNA (-Id4, vector A). **C**: Immuno-cytochemical analysis of stable knockdown of Id4 expression in LNCaP cells (LNCaP-Id4, vector A) as compared to cells with non-specific shRNA (LNCaP + NS). The red staining indicates Id4 expression (DyLight 594). Id4 expression in DU145 cells stably transfected with Id4 expression vector (DU145 + Id4) as compared to DU145 cells transfected with empty vector (DU145 + NS). The green staining represents Id4 (DyLight 488). DAPI was used to stain the nuclei (blue) in both LNCaP and DU145 cells. Representative images are shown.

Id4 is undetectable in DU145 cells due to promoter hyper-methylation [18]. In contrast, Id4 is expressed in LNCaP cells. These two cell lines were used to either over-express (DU145 + Id4) or silence (LNCaP-Id4) Id4. Three different retroviral shRNA vectors (vectors A, B and C) were used to silence Id4 (Figure 1, vector B had no effect on Id4 levels, not shown) in LNCaP cells. The stable knockdown of Id4 in LNCaP cells using shRNA vector A (LNCaP-Id4), Id4 over-expressing DU145 cells (DU145 + Id4, Figure 1C) and their respective vector only transfected cells were used for all subsequent experiments.

### Id4 promotes apoptosis

**Figure 2:**
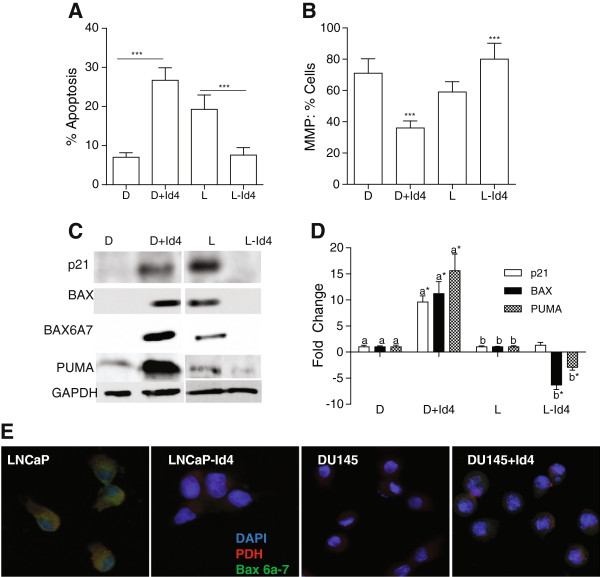
**Id4 promotes apoptosis by regulating mitochondrial membrane potential and the expression of pro-apoptotic genes. A**: percent cells undergoing apoptosis was determined by propidium iodide and Annexin V staining followed by flow cytometery. Significant increase in apoptosis (***: P < 0.001) was observed in DU145 cells over-expressing Id4 (D + Id4) when compared with DU145 cells alone **(D)**. A significant decrease in apoptosis was observed in LNCaP cells that lacked Id4 (L-Id4) as compared to LNCaP cells (L, ***: P < 0.001). **B**. Percent cells with high mitochondrial membrane potential (Gated, FL2 > 100 fluorescence units). In the presence of Id4 (D + Id4 and L), the mitochondrial membrane potential decreased as compared to the corresponding cells that lack Id4 (D and L-Id4). (***: P < 0.001–L vs. L-Id4 and D vs. D + Id4). **C**. Western blot analysis of p21, BAX, conformation specific BAX (BAX6A7) and PUMA in D, D + Id4, L and L-Id4 cells. GAPDH was used as loading control. Representative western blots of three different experiments are shown. **D**. Real time quantitative analysis of p21, BAX and PUMA expression in D, D + Id4, L and L-Id4 cells. The mean ± SEM of three experiments in triplicate is shown. The ΔΔCt (normalized to GAPDH) between D and D + Id4 (D normalized to 1, designated as “a”) and between L and L-Id4 (L normalized to 1, designated as “b”) is shown (*: P < 0.001). **E**. Immuno-cytochemical analysis demonstrating co-localization of conformation specific BAX (using BAX 6A7 antibody) with mitochondrial pyruvate dehydrogenase (PDH). Blue: DAPI, red: PDH, green: BAX 6A7 and yellow: co-localization of BAX and PDH (observed only in LNCaP and DU145 + Id4 panels. Representative images from three different experiments are shown.

A significant increase in apoptotic cells (Annexin V positive) was observed in DU145 + Id4 (26.7 ± 3.2%, P < 0.001, Figure 2A) cells as compared DU145 cells (7.1 ± 1.2%, Figure 2A) whereas number of cells undergoing apoptosis decreased in LNCaP-Id4 (7.6 ± 1.9%) as compared to LNCaP (19.3 ± 3.6%) cells (Figure 2A). Apoptosis in DU145 + Id4 cells was accompanied by decreased mitochondrial membrane potential (MMP, 36 ± 4.94%, Figure 2B) whereas decreased apoptosis in LNCaP-Id4 cells was associated with increased MMP (82.3 ± 10.21%) as compared to DU145 (71.3 ± 9.30%) and LNCaP (59.4 ± 6.60%) respectively (Figure 2B). These results led us to conclude that Id4 promotes apoptosis through changes in MMP that eventually promotes cytochrome c release from the mitochondria [35].

Increased BAX expression and/or PUMA dependent dissociation of BAX from Bcl-2 promotes translocation of BAX to mitochondria resulting in decreased mitochondrial membrane potential [36]. The expression of pro-apoptotic BAX and PUMA increased in DU145 + Id4 cells whereas a corresponding decrease in BAX and PUMA was observed in LNCaP-Id4 cells at the protein (Figure 2C) and transcript (Figure 2D) level as compared to DU145 and LNCaP cells respectively (Figure 2C and D). These results implicated the role of Id4 in promoting apoptosis through increased expression of BAX and PUMA. Activation of BAX in response to apoptotic stimuli is characterized by translocation and multimerization on the mitochondrial membrane surface resulting in exposure of an amino terminal epitope recognized by the conformation specific monoclonal antibody BAX 6A7 [37]. Co-localization of BAX (BAX 6A7 antibody) with mitochondrial PDH (pyruvate dehydrogenase) demonstrated that BAX undergoes conformational change and translocates to the mitochondria in DU145 + Id4 and LNCaP cells (Figure 2E) but not in DU145 and LNCaP-Id4 cells possibly due to undetectable levels of BAX (Figure 2C).

Next, we investigated the expression of CDKN1A (p21) which is also a well-characterized p53 responsive gene [38]. The p21 protein and transcript expression increased significantly in DU145 + Id4 cells as compared to DU145 (Figure 2C and D, 9 fold as compared to DU145). The p21 protein expression in LNCaP-Id4 cells also decreased as compared to LNCaP, but intriguingly the levels of p21 transcript (mRNA) were similar between LNCaP-Id4 and LNCaP cells.

### Id4 alters expression and cellular localization of p53

**Figure 3:**
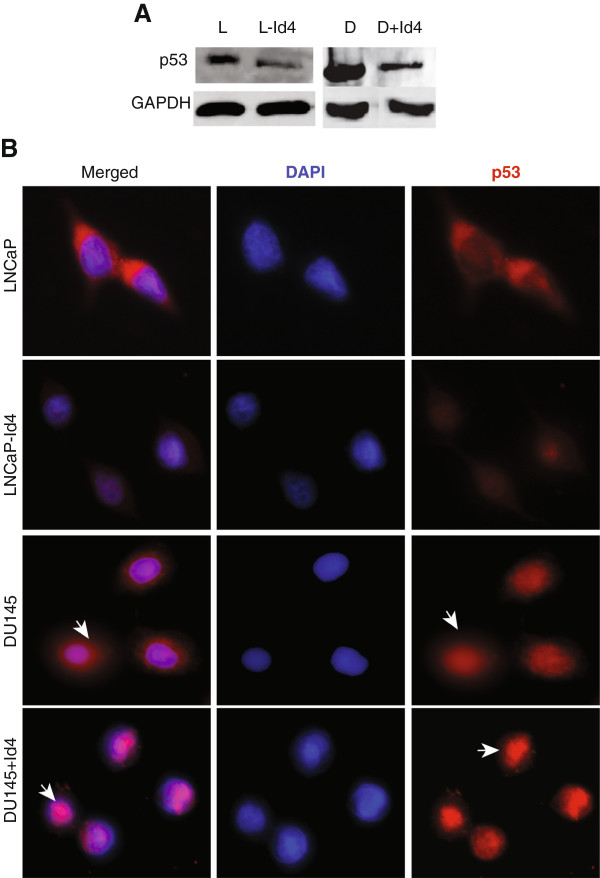
**Id4 regulates p53 expression and cellular localization.** Analysis of p53 protein **(A)** expression in L, L-Id4, D and D + Id4 cells. The western blot analysis shown in panel **A** is the representative of three different experiments. **B**. Immuno-cytochemical localization of p53 in L, L-Id4, D and D + Id4 cells. Nuclear and cytoplasmic (arrows) expression of p53 is clearly evident in L, L-Id4 and D cells. Whereas p53 is primarily nuclear in D + Id4 cells (arrows). Red: p53, Blue: DAPI. Representative images are shown.

Both BAX and PUMA are also transcriptional targets of the tumor suppressor protein p53 [39]. Reduced apoptosis in part due to loss of BAX and PUMA expression in LNCaP-Id4 cells was associated with low p53 expression as compared to LNCaP cells (Figure 3A). A similar relationship between Id4 and p53 expression was not observed in DU145 cells. Unlike wt-p53 in LNCaP cells, the DU145 cells harbor a mutant p53 (mut-p53). The two mutations (P223L and V274F) are within the DNA binding domain resulting in a transcriptionally inactive form of p53 [22]. Mut-p53 protein generally accumulates at high levels due to loss of regulatory mechanisms [40] as seen in DU145 cells (Figure 3A and B, 12 fold higher as compared to LNCaP cells). Surprisingly, we observed decreased levels of mut-p53 in DU145 + Id4 cells (Figure 3A). These results are significant especially in context of increased expression BAX and PUMA in DU145 + Id4 cells in spite of low mut-p53 expression. We reasoned that one of the mechanisms by which mut-p53 could up-regulate BAX/PUMA expression could be through gain of transcriptional activity in DU145 + Id4 cells. Immuno-cytochemical localization of p53 also revealed that mut-p53 is localized to the nucleus and cytoplasm in DU145 (Figure 3B, DU145, arrows) cells but is primarily nuclear in DU145 + Id4 cells (Figure 3B, DU145 + Id4, arrows). Previous studies have also shown a predominant cytoplasmic staining of mutant p53 in prostate cancer whereas wt-p53 is primarily nuclear [41].

### Id4 restores mutant p53 DNA binding and transcriptional activity

**Figure 4:**
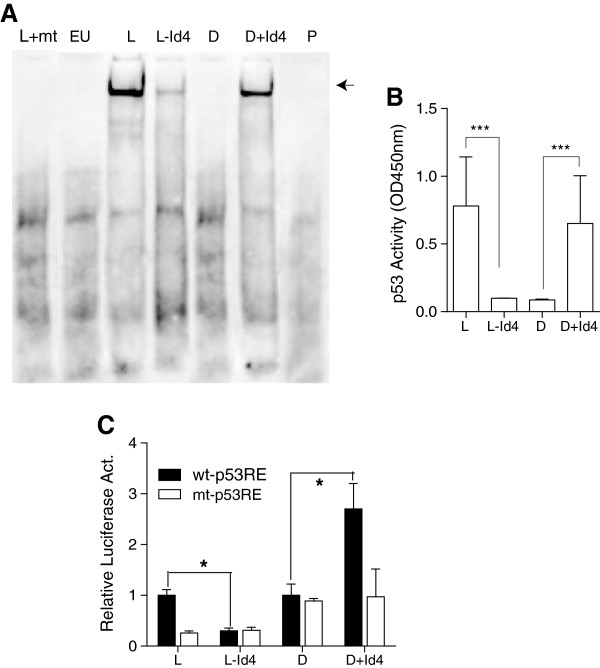
**Id4 promotes DNA binding and transcriptional activity of wild type and mutant p53. A**. EMSA with p53 consensus DNA binding response element with nuclear extracts from LNCaP (L), LNCaP-Id4 (L-Id4), DU145 (D), DU145 + Id4 (D + Id4) and PC3 cells. Nuclear extracts from PC3 cells, null for p53 and LNCaP cells with wild type p53 were used as negative and positive controls respectively for binding to wild type p53 response element. Excess unlabeled (EU) wild type p53 response element was used to monitor non-specific binding. The biotin labeled mutant p53 response element (mt) incubated with nuclear extracts from LNCaP cells (L + mt) was used to demonstrate specificity of EMSA. **B**. Quantitative p53 DNA binding in a sandwich ELISA based system. P53 was captured by double stranded oligonucleotide with p53 response element immobilized on a 96 well plate. The captured p53 was detected using p53 antibody by measuring the intensity at 450 nm using HRP coupled secondary antibody. **C**. The p53 transcriptional activity as determined by transiently transfecting cell lines as indicated above with p53 response element driven luciferase reported plasmid (wt-p53RE). The data is normalized to Renilla luciferase. The mutant p53 luciferase reporter plasmid was used as a negative control (mt-p53RE). The p53-luciferase reporter activity in LNCaP-Id4 (L-Id4) was normalized to LNCaP (L) and that of DU145 + Id4 (D + Id4) with DU145 (D). The data from 3 different experiments in triplicate is expressed as mean + SEM (*: P < 0.001).

An EMSA with canonical p53 DNA response element was used to determine the DNA binding ability of wt-(LNCaP) and mut-p53 (DU145). LNCaP cells with wt-p53 resulted in a gel shift (Figure 4A), whereas a gel shift of lower intensity was observed in LNCaP-Id4 as compared to LNCaP cells perhaps due to lower expression of wt-p53 (Figure 3A and B). A distinct gel shift was observed in the presence of DU145 + Id4 nuclear extracts, but no gel shift was observed with DU145 nuclear extracts, suggesting that mut-p53 in the absence of Id4 lacks DNA binding activity. Increased binding of p53 to its cognate response element immobilized on a 96 well plate followed by detection with p53 specific antibody was also observed in LNCaP and DU145 + Id4 that was significantly higher as compared to LNCaP-Id4 and DU145 cells respectively (Figure 4B). In a functional transcriptional assay using a p53 response element (wt-p53RE) luciferase reporter plasmid, the relative p53 luciferase activity decreased significantly in LNCaP-Id4 cells as compared to LNCaP cells (normalized to 1, Figure 4C), which is consistent with the expression of p53 in these cell lines. Surprisingly, mut-p53 in DU145 + Id4 cells demonstrated high luciferase activity as compared to DU145 (normalized to 1, wt-p53RE). The mutant p53 luciferase plasmid (mt-p53RE) used as a negative control, as expected, did not result in significant luciferase activity. In context of using LNCaP as a positive control, our results strongly suggested that mut-p53 gains DNA binding and transcriptional activity in the presence of Id4 that is in part independent of its expression level. Silencing of p53 through siRNA was used to further clarify the role of mutant p53 in DU145. However, siRNA based p53 silencing led to massive apoptosis in DU145 [42].

### Id4 enhances p53 binding to target promoters

**Figure 5:**
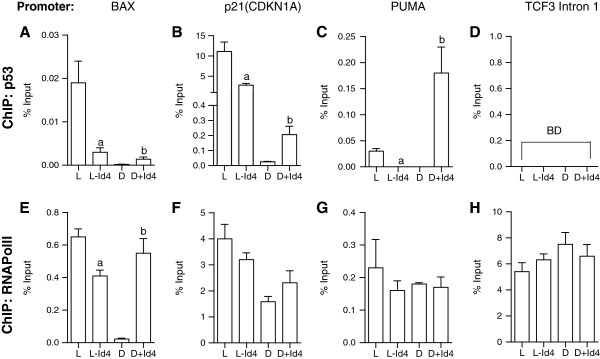
**Chromatin immuno-precipitation assay demonstrating the enrichment of p53 (A, B, C and D) and RNA polymerase II (RNA Pol II, E, F, G and H) on the BAX, p21 and PUMA promoters.** The intron 1 region of TCF3 gene was used as a negative control for p53 ChIP studies (D). The data is expressed as percent input is mean ± SEM of three experiments in triplicate (a: between L and L-id4 and b: between D and D + Id4, *: P < 0.001, BD: Below Detection)

Real time quantitative PCR analysis on Chromatin immuno-precipitated (ChIP) DNA with p53 antibody demonstrated the binding of wt-p53 to its respective response elements on BAX (Figure 5A), p21 (Figure 5B) and PUMA (Figure 5C) promoters in LNCaP cells. The enrichment of p53 on the respective promoters (p21, BAX and PUMA) was specific since we did not observe a similar enrichment on intron 1 of TCF3 gene that lacks a consensus p53 response element as determined TRANSFAC database search [31] (Figure 5D). The decreased p53 expression in LNCaP-Id4 correlated with decreased binding to its respective promoter elements on BAX, p21 and PUMA promoters (P < 0.001). As anticipated, in DU145 cells no significant binding of mutant p53 was observed on p21, PUMA and BAX promoters. However, in DU145 + Id4 cells, a significant increase in the binding of mut-p53 as compared to DU145 cells was observed on BAX, p21 and PUMA promoters.

RNA polymerase II (Pol II) was constitutively bound to the PUMA (Figure 5G) and p21 promoters (Figure 5F) in LNCaP and LNCaP-Id4 cells lines suggesting that binding of p53 was required to initiate transcription form these promoters but not for the assembly of the transcription pre-initiation complex. On BAX promoter, a significant decrease in the enrichment of RNA Pol II promoter was observed in LNCaP-Id4 cells as compared to LNCaP cells, whereas a significantly higher enrichment of RNA Pol II was observed in DU145 + Id4 cells as compared to DU145 cells (Figure 5E). These results suggested that binding of p53 may be required for recruitment RNA Pol II complex on BAX promoter in these two cell lines.

### Id4 promotes p53 dependent MDM2 expression

**Figure 6:**
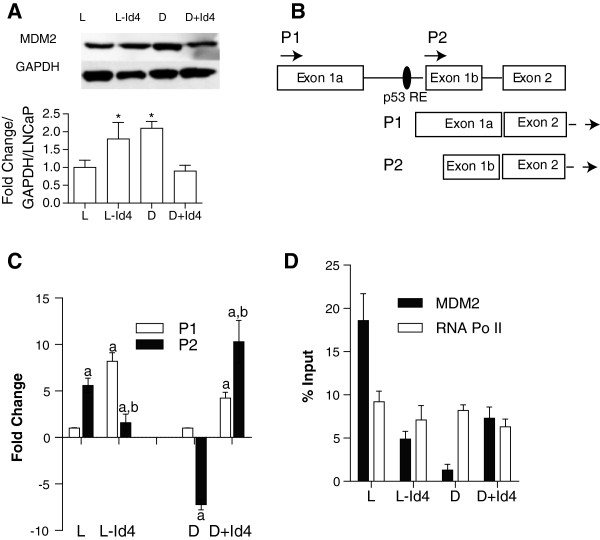
**Expression of MDM2 and its transcriptional regulation. A**. MDM2 immuno blot in cells with (L and D + Id4) and without Id4 (L-Id4 and D). GAPDH was used as loading control. Representative data from three different experiments is shown. The bottom panel is semi-quantitative analysis of fold change in MDM2 expression relative to LNCaP (L) and normalized to GAPDH (mean ± SEM, *: P < 0.001, compared to L). **B**. Schematic of MDM2 promoter organization. MDM2 is transcribed from two independent promoters P1 and P2 but both the transcripts are translated from a common start site in exon2. P1 promoter is p53 independent whereas P2 promoter is p53 dependent due to a p53 response element in intron 1 (p53RE). Specific primers were used to determine the transcript abundance of P1 (p53 independent) and P2 (p53 dependent) transcripts. **C**. P1 and P2 transcript abundance with Real time quantitative PCR analysis in cell lines expressed as fold change from three different experiments in triplicate (mean ± SEM). The expression is first normalized to GAPDH and then to P1 transcript in L and D cells set to 1 (comparison between L and L-Id4 and between D and D + Id4, a: P < 0.001 as compared to P1 transcript b: P < 0.001 compared to P2 transcript). **D**. Chromatin immuno-precipitation assay demonstrating the binding of p53 to its respective response element in the MDEM2 P2 promoter (intron 1). Data is expressed as mean + SEM of three different experiments performed in triplicate (mean + SEM, *: P < 0.001).

Incidentally, p53 also regulates MDM2, (an E3 ubiquitin ligase involved in p53 protein degradation) expression in a highly complex manner. In this study we focused on investigating whether MDM2 expression is regulated in a p53 dependent manner at the promoter level, rather than on interaction between wt- and mut-p53 with MDM2 at the protein level. Unpredictably, MDM2 protein expression was higher in LNCaP-Id4 (1.8 ± 0.46 fold, Figure 6A) cells as compared to LNCaP cells (Figure 6A and semi quantitation in lower panel) in spite of lower p53 expression (Figure 3A and B). The expression in DU145 cells (2.1 ± 0.19 fold) was comparable to LNCaP-Id4 cells (Figure 6A). However, MDM2 expression was lower in DU145 + Id4 (0.9 ± 0.16) cells as compared to DU145 but was comparable to LNCaP cells (normalized to 1). MDM2 expression is regulated by a p53 response element located within the P2 promoter in intron 1 (Figure 6B) [43]. The alternative, P1 promoter, upstream of exon1 is generally considered p53 independent [44]. Both P1 and P2 transcripts are however translated from the common start site in exon 2. Abundance of P1 and P2 transcripts was then performed to understand whether MDM2 expression is regulated in a p53 dependent (P2) or independent (P1) manner. The results suggested that MDM2 expression in LNCaP cells is primarily due to transcription from the P2 promoter in part due to the binding of p53 (Figure 6D), whereas in LNCaP-Id4 cells, MDM2 expression is a result of activation from the P1 promoter (Figure 6C). In DU145 cells, the P1 promoter was active as compared to P2, but in DU145 + Id4 cells, the p53 dependent (Figure 6D) P2 promoter was transcriptionally active (Figure 6C). These results suggested that the regulation of MDM2 expression is highly complex and that in cells lacking Id4 (LNCaP-Id4 and DU145), the P1 promoter is transcriptionally active whereas in cells with Id4 (LNCaP and DU145 + Id4) the p53 dependent P2 promoter is active (Figure 6D).

### Id4 Recruits CBP/p300 to promote p53 acetylation

**Figure 7:**
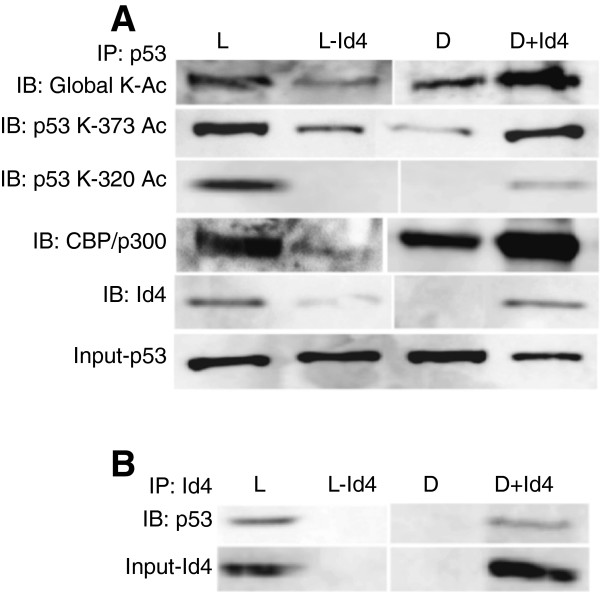
**Acetylation of p53 and interaction with CBP/p300 and Id4. A**. p53, immuno-precipitated from cell lines was blotted with antibodies against acetylated lysine (global), p53 acetylated at either K373 (Ac-373) or K320 (Ac-320), CBP/ p300 and Id4. **B**. The total protein lysate from cell lines as indicated was immuno-precipitated (IP) with Id4 antibody. The immuno-precipitated lysate was then immuno-blotted with p53 antibody (IB: p53). Representative data is shown.

Acetylation, independent of phosphorylation status, promotes p53 stabilization and transcriptional activity but de-stabilizes its interaction with MDM2 [45]. Recent studies have also shown that acetylation of some mutant forms p53 can restore the DNA binding activity [26]. These studies led us to explore whether Id4 promotes acetylation of mut-p53 in DU145 + Id4 cells. The total p53 protein was first immuno-precipitated and then immuno-blotted with acetylated lysine antibody. Increased global p53 lysine acetylation was observed in DU145 + Id4 and LNCaP cells as compared to LNCaP-Id4 and DU145 cells (Figure 7A). In p53, K320 is acetylated by PCAF and promotes p53-mediated activation of cell cycle arrest genes such as p21 [46]. In contrast, acetylation of K373 leads to hyper-phosphorylation of p53 NH_2_-terminal residues and enhances the interaction with promoters for which p53 possesses low DNA binding affinity, such as those contained in pro-apoptotic genes, BAX and PUMA [46]. The results shown in Figure 7A demonstrated a significant increase in K373 acetylation in DU145 + Id4 cells whereas no significant change was observed between LNCaP and LNCap-Id4 cells. The K320 expression was also significantly higher in DU145 + Id4 and LNCaP cells as compared to DU145 and LNCaP-Id4 cells. These results provided evidence that Id4 is involved in promoting acetylation of specific residues in wt-and mut-p53 that promotes its binding to respective response elements. The increased K320 acetylation in DU145 + Id4 cells clearly is consistent with the study by Parez et al. [26] in which the authors demonstrated acetylation at this specific residue restores mutant p53 biological activity. We were however intrigued with a significant increase in the expression of acetylated K373 in DU145 + Id4 cells. Acetylation at K373 is CBP/P300 dependent [47]. We hypothesized that if CBP/P300 is involved in K373 acetylation then it could co-precipitate with p53. Results demonstrated that indeed mutant p53 is physically associated with CBP/P300 in DU145 + Id4 cells at significantly higher levels than mut-p53 from DU145 cells alone (Figure 7A). These results led us to propose a model whereby Id4 could recruit or promote the assembly of CBP/P300 and p53.

### Id4 Interacts with p53

Immuno-precipitation with Id4 and blotting with p53 demonstrated the presence of p53 in this complex in DU145 + Id4 and LNCaP cells but not in DU145 and LNCaP-Id4 cells suggesting that Id4 directly associates with p53 (Figure 7B). Id4 was also co-eluted with p53 (Figure 7A) which confirms the specificity of this interaction and further supports the formation of a large multi-protein complex involving Id4, CBP/p300 and p53. These results consolidated our hypothesis that Id4 promotes the recruitment of CBP/p300 on p53 to promote acetylation and restore its biological activity.

## Discussion

In this study we provide evidence that Id4 regulates p53 at two different levels: transcriptional regulation of wt-p53 in LNCaP cells and restoration of the biological activity of mutant p53 in DU145 cells. In this study, we focused on investigating the mechanism by which Id4 restores the biological activity of mutant p53, clearly an area of high interest given that mutant p53 is observed in one third of prostate cancer [27,28] and more than 50% of all cancers [48]. The down-regulation of wt-p53 protein expression in the absence of Id4 in LNCaP cells (LNCaP-Id4) is a significant observation that was not addressed in this study. We speculate that Id4 could interact and modify the transcriptional regulators of p53 expression which remains to be investigated.

The core domain (aa 98-303) of p53 is inherently unstable. Point mutations in this domain promote instability and unfolding, leading to decreased or completely abrogated transcriptional activity [49]. Both the alleles of p53 in DU145 cells (p223L and V274F) carry mutations within this core domain resulting in increased expression of mutant p53 [22] with predominantly denatured conformation. The attenuated transactivation potential of p53 P223L and V274F mutants is also observed when over-expressed in p53 null PC3 cells [50]. Hence the mutants in DU145 cells are excellent models to understand the mechanisms involved in promoting its function in context of Id4 which is epigenetically silenced in DU145 cells.

In our studies we clearly show high mutant p53 expression in DU145 cells with attenuated transactivation potential and DNA binding activity as compared to LNCaP cells with wt-p53. Multiple lines of evidence support the gain of transactivation potential of mutant p53 in DU145 cell over-expressing Id4: First, mutant p53 in DU145 + Id4 cells promotes p53 dependent luciferase reporter activity, second, mutant p53 gains DNA binding activity as demonstrated by EMSA and direct DNA binding followed by detection and quantitation of binding with p53 specific antibody and thirdly, site specific binding to the respective p53 binding sites on BAX, PUMA, p21 and MDM2 P2 promoters. Studies have also shown that virtually all tumor derived p53 mutants are unable to activate BAX transcription but some retain the ability to activate p21 transcription [25]. However, our results suggest the p53 mutations in DU145 are incapable of trans-activating not only p21 but BAX as well due to lack of promoter binding. The decrease in the expression of mutant p53 in DU145 + Id4 cells as compared to DU145 could also suggest that mutant p53 responds to the regulatory network required to maintain its normal physiological (compared to LNCaP cells) levels that needs to be investigated. The post-translation modifications within p53 (discussed below) can promote its function at multiple levels by attenuating its interaction with MDM2, recruitment to p53 responsive promoters and favoring nuclear retention as observed in DU145 + Id4 cells.

The discrepancy between p21 expression at the transcript and protein level was also observed in LNCaP-Id4 cells. The amount of p53 bound to the respective response element and RNA pol II, especially on the p21 promoter is not the sole determinant of transcriptional repression [39] as seen in LNCaP-Id4 cells, in which p21 transcript abundance is not significantly different from LNCaP cells. A significant decrease in p21 protein expression in LNCaP-Id4 cells could be due to increased proteolysis. Increased MDM2 expression in LNCaP-Id4 could facilitate the binding of p21 with the proteosomal C8-subunit [51] in a ubiquitin independent manner. Alternatively, loss of Id4 may promote proteolysis of p21 through ubiquitin dependent mechanisms involving e.g. Skp1/cullin/F-box (SCF) complexes that remain to be investigated (reviewed in [52]).

Acetylation at lysine residues has emerged as a critical post-translational modification of p53 for its function *in vivo* such as growth arrest, DNA binding, stability and co-activator recruitment ( [45,46] and reviewed in [53]). The global de-acetylation of p53 and specifically at K320 and K373 in LNCaP-Id4 cells provide strong evidence that acetylation is a major modification required to maintain wild type p53 activity. Our results on mutant p53 acetylation, global and K320/ 373 specific in DU145 + Id4 are particularly novel and provide direct evidence that mutant p53 activity can be restored by acetylation. The increased K320 acetylation of DU145 p53 mutants is most likely also mediated by PCAF but we did not directly investigate this mechanism. However, a significant observation made in this study was co-elution CBP/P300 with wt-(LNCaP) and mutant p53 (DU145 + Id4) and increased K373 acetylation in an Id4 dependent manner. Moreover, co-elution of Id4 as part of this complex with p53 antibody and co-elution of p53 with Id4 antibody suggest that Id4 can recruit CBP/P300 on wt-and mutant p53 to promote acetylation. Alternatively, CBP/p300 could recruit Id4 to promote large macromolecular assembly on p53 that could result in its acetylation and increased biological activity. Thus certain p53 mutations with some degree of conformational flexibility, upon co-factor recruitment such as Id4 and CBP/p300 could gain biological activity that is similar to wt-p53.

Acetylation at specific lysine residues can also promote specific p53 functional modifications: acetylation at K320 by PCAF results in increased cytoplasmic levels whereas CBP/P300 dependent acetylation of K370/372/373 leads to increased nuclear retention of p53 [46,47]. In contrast, MDM2, a negative regulator of p53, actively suppresses p300/CBP-mediated p53 acetylation *in vivo* and *in vitro*[54]. In this study we did not investigate the role of phosphorylation in regulating wt-or mut-p53 activity. K373 acetylation mimic p53Q373 undergoes hyper-phosphorylation and interacts more strongly with low affinity pro-apoptotic promoters such as BAX. In contrast, the p53Q320 interacts efficiently with the high-affinity p21 promoter [46]. The ChIP data demonstrating high p53 binding on p21 promoter in DU145 + Id4 cells with increased p53 K320 acetylation may suggest increased phosphorylation that correlates well and further supports acetylation dependent increase in mutant p53 activity.

As such, low MDM2 levels observed in DU145 + Id4 cells as compared to DU145 could be one of the mechanism by which mutant p53 could gain its trans-activation potential together with increased acetylation. MDM2 binds to the N-terminal end of p53 to inhibit its trans-activation function partly by suppressing p300/CBP-mediated p53 acetylation [54]. Acetylation also destabilizes p53-MDM2 interaction and enables p53 mediated response including recruitment to respective promoters and apoptosis [38]. Studies in DU145 and LNCaP cells using nutlin, a disruptor of p53-MDM2 interaction, suggested that blocking MDM2 interaction or decreasing its cellular levels may be sufficient to promote wt-p53 activity (LNCaP cells) but is not sufficient for promoting mutant p53 transcriptional activity in DU145 cells [21].

In a recent study [55], Id4 expression was shown to be regulated by mutant p53 in an E2F1 dependent manner in breast cancer cell lines SKBR3 (p53 R175H) and MDA-MB-231 (p53 R280K). Both these cell lines were also shown to express Id4 [55]. Meta-analysis on clinical samples revealed that mutant p53 breast cancer tumors under-express Id4 suggesting an inverse correlation [56] as seen in DU145 cells. Based on our results, we speculate that in the study by Fontemaggi et al., [55] Id4 could restore functional conformation of mut-p53, by acetylation in breast cancer cell lines leading to increased transcriptional activity. The mut-p53 in SKBR3 cells can be restored to functional conformation by Zinc [57] further suggesting that mut-p53 retains the flexibility to undergo functional conformation to mimic wild type p53 activity.

## Conclusions

We provide evidence that mutant p53 in DU145 cells retains the ability to undergo acetylation in the presence of Id4. Id4, a transcriptional regulator, may promote the p53 acetylation by recruiting CBP/p300 and/or PCAF, independent of p53 mutations. Acetylated p53 in turn acquires transcriptional activity through structural changes that could possibly involve destabilization of p53-MDM2 interaction, and subsequent recruitment to p53 responsive genes and promote apoptosis. The global acetylation promoted by Id4 suggests that additional lysines such as K120 and K164, known to increase binding to specific p53 responsive genes such as PUMA could also be involved, but remains to be investigated. Whether Id4 promotes the activity of p53 mutants found only in DU145 cells or it has the ability to promote transactivation potential of other well-known p53 hot-spot mutants is an obvious next step that needs to be investigated. Nevertheless, the acetylation mechanism is nearly universal in nature and suggests that Id4 could promote the biological activity of other mutants, however whether such mutants retains sufficient structural flexibility following acetylation remains to be determined. Our results also suggest that Id4 regulates the activity of wild type p53, a significant observation that requires further validation in other cell types.

## Competing interests

The authors declare that there is no competing financial interest in relation to the work described.

Financial competing interests: Authors declare no financial competing interests.

## Authors’ contributions

AEK: developed the L-Id4 cell lines, ChIP experiments, Immuno-blot studies, qRT-PCR, First draft of the manuscript. DP: Apoptosis assays, Mitochondrial membrane potential, ChIP experiments, immuno-blots, luciferase assays. DJM: Immuno-blots, p53 DNA binding ELISA. PS: Immuno-precipitations and Gel shift assays. SG: Immuno-cytochemistry. JC: Conceived the study and final draft of the manuscript. All authors read and approved the final manuscript.

## Supplementary Material

Additional file 1: Table A1 Antibodies and reagents for Immuno blots, Immuno-cytochemistry and CHiP. Click here for file

Additional file 2: Table A2 qRT-PCR and CHiP primers used in the study. Click here for file

## Support

The work was supported by NIH/NCI CA128914 (JC) and in part by NIH/NCRR/RCMI G12RR03062.
